# Single-structure 3-axis Lorentz force magnetometer based on an AlN-on-Si MEMS resonator

**DOI:** 10.1038/s41378-024-00696-3

**Published:** 2024-05-09

**Authors:** Cheng Tu, Xu-heng Ou-Yang, Ying-jie Wu, Xiao-sheng Zhang

**Affiliations:** School of Integrated Circuit Science and Engineering, University of Electronic Science and Technology of China, Chengdu, 611731 China

**Keywords:** Electrical and electronic engineering, Sensors, Electronic devices

## Abstract

This work presents a single-structure 3-axis Lorentz force magnetometer (LFM) based on an AlN-on-Si MEMS resonator. The operation of the proposed LFM relies on the flexible manipulation of applied excitation currents in different directions and frequencies, enabling the effective actuation of two mechanical vibration modes in a single device for magnetic field measurements in three axes. Specifically, the excited out-of-plane drum-like mode at 277 kHz is used for measuring the x- and y-axis magnetic fields, while the in-plane square-extensional mode at 5.4 MHz is used for measuring the z-axis magnetic field. The different configurations of applied excitation currents ensure good cross-interference immunity among the three axes. Compared to conventional capacitive LFMs, the proposed piezoelectric LFM utilizes strong electromechanical coupling from the AlN layer, which allows it to operate at ambient pressure with a high sensitivity. To understand and analyze the measured results, a novel equivalent circuit model for the proposed LFM is also reported in this work, which serves to separate the effect of Lorentz force from the unwanted capacitive feedthrough. The demonstrated 3-axis LFM exhibits measured magnetic responsivities of 1.74 ppm/mT, 1.83 ppm/mT and 6.75 ppm/mT in the x-, y- and z-axes, respectively, which are comparable to their capacitive counterparts.

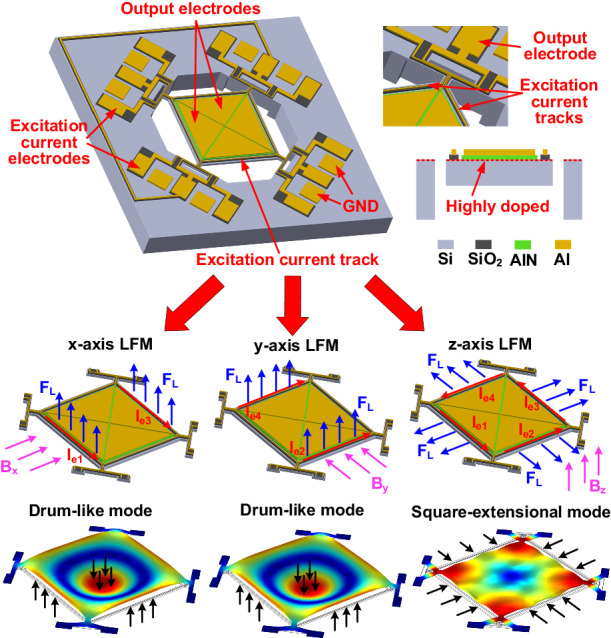

## Introduction

With the rapid advancement of microfabrication techniques, microelectromechanical system (MEMS) Lorentz force magnetometers (LFMs) have become a promising alternative to common technologies, such as Hall-effect sensors and magnetoresistive (MR) sensors^1,2^. One advantage of MEMS LFMs is their fabrication compatibility with complementary metal‒oxide‒semiconductor (CMOS) electronics, which enables monolithic integration of LFMs and signal conditioning circuits, thus reducing their footprints, parasitic elements, power consumption levels and overall manufacturing costs^3^. Moreover, MEMS LFMs have attracted considerable attention because they can be fabricated together with micromachined accelerometers, gyroscopes and pressure sensors using the same microfabrication process, which allows the possibility of realizing single-chip multi-degree-of-freedom inertial measurement units (IMUs)^4^. Generally, MEMS LFMs utilize micromechanical resonators actuated or perturbed by the Lorentz force resulting from the cross product of external magnetic field and excitation current. Thus, the strength of the magnetic field can be deduced by measuring the change in either vibration amplitude or resonant frequency of the resonator. The former refers to amplitude modulation (AM) mode while the latter refers to frequency modulation (FM) mode. Reportedly, AM-mode LFMs generally show greater sensitivity than FM-mode LFMs^5^, although the latter feature quasi-digital output, which improves the immunity to interference. According to the transduction scheme, MEMS LFMs can be classified into different types, such as capacitive, piezoelectric and thermal-piezoresistive LFMs. Capacitive LFMs require vacuum sealing to reduce the viscous damping resulting from the very small capacitive gap and thus increase the mechanical quality factor (Q)^6^. In contrast, piezoelectric LFMs are able to operate with high Q values at ambient pressure, which significantly relieves the burden of vacuum encapsulation^7^. Another advantage of piezoelectric LFMs is their much larger electromechanical transduction efficiency compared to that of capacitive LFMs, which helps reduce the complexity of interface circuit design^8^. Thermal piezoresistive LFMs can also operate at ambient pressure due to their strong electromechanical coupling enabled by the internal Q-amplification effect^9^. However, there is a concern about power consumption and thermal noise for this type of LFM.

During the past decade, there has been a growing demand for single-structure 3-axis magnetometers since they are more area-efficient and consume less power during assembly than three discrete magnetometers, each of which is responsible for measuring the magnetic field in a single axis. In response to this demand, much work has been performed regarding single-structure 3-axis capacitive LFMs^10–12^. However, single-structure 3-axis LFMs using piezoelectric transduction have not yet been reported. The difficulty in realizing single-structure 3-axis piezoelectric LFMs lies in the fact that a fixed current path cannot generate a Lorentz force on all three orthogonal magnetic fields. Furthermore, the generated Lorentz forces of a current path in response to orthogonal magnetic fields are in different directions. This suggests that a single mechanical vibration mode or fixed current path cannot fulfill the function of 3-axis magnetic field measurement. Another issue for piezoelectric LFMs is the lack of an equivalent circuit model that can be used to help design and analyze such devices. This work reports the first single-structure 3-axis piezoelectric LFM with an equivalent circuit model, which can correlate the measured output currents with the applied magnetic fields. The functionality of the 3-axis measurement is realized by reconfiguring both the current paths and mechanical vibration modes of a square-plate AlN-on-Si resonator operating in AM mode. The advantage of using the AlN-on-Si structure as a resonating sensor is twofold. First, a thin AlN film, as a piezoelectric transducer, exhibits decent electromechanical coupling and is compatible with post-CMOS processing due to its low deposition temperature ( < 400 °C)^13^. Second, acoustic microresonators composed of single-crystal silicon show very low acoustic damping with superior mechanical robustness^14^. Thus, AlN-on-Si resonators usually offer the benefits of low motion resistance, high Q and fabrication compatibility with CMOS circuitry. Notably, AlN-on-Si resonators have already been successfully used to construct resonant accelerometers, gyroscopes, pressure sensors and thermometers^15–18^, which, together with LFMs, promise a monolithic integration solution for piezoelectric IMUs.

## Working mechanism

### Device description

**Fig. 1:**
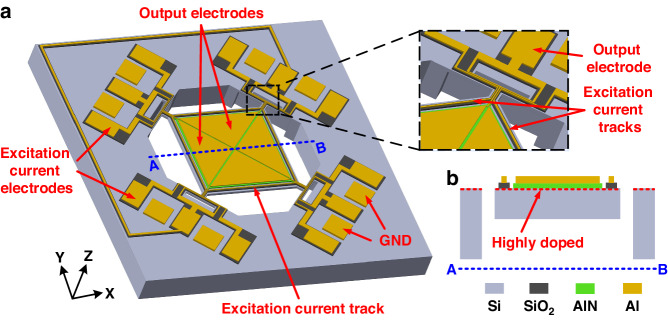
Schematic of the proposed single-structure 3-axis LFM based on a square-plate AlN-on-Si resonator. **a** Perspective-view schematic. **b** Cross-sectional view schematic of the device as seen across the AB cutline

Figure 1a shows a perspective-view schematic of the proposed single-structure 3-axis LMF, which comprises a square-plate AlN-on-Si resonator. There are four triangular output electrodes on the plate with two electrodes connected diagonally on the chip, forming a two-port configuration. Given that the two target vibration modes both yield charges with the same polarity at four output electrodes, all electrodes are electrically connected as a single output port when the device is used as an LFM operated in an open-loop scheme. Note that the two-port electrode configuration is designed to allow the realization of a closed-loop scheme. There is an Al track running along each edge of the square plate, providing an electrical path for the excitation current. The excitation current is applied through two adjacent excitation current electrodes. The square plate resonator is suspended by four T-shape anchors at the corners, as shown in the inset of Fig. 1a. The use of a T-shape anchor instead of a straight-beam anchor aids in effectively exciting the two target mechanical vibration modes. There are three Al tracks on the T-shape anchor: the tracks on two sides provides electrical paths for excitation currents, and the middle track provides the path for output current. In such a configuration, four excitation currents at four edges can be manipulated individually, which allows flexible control of the Lorentz force generated at four edges toward the two target vibration modes. Figure 1b shows a cross-sectional view of the device at cut-line AB. An AlN layer is sandwiched between the output electrode and highly doped silicon plate to form a piezoelectric transducer. As the square-plate resonator is driven into vibration by the Lorentz force, the generated charges are collected at the output electrodes due to the direct piezoelectric effect. Note that a SiO_2_ layer, instead of an AlN layer, is used to isolate the excitation current track from the underlying highly doped silicon plate to avoid unwanted piezoelectric actuation directly from the excitation current.

A similar square-plate structure has been reported previously for measuring the z-axis magnetic field, which utilizes two separate excitation current tracks mirrored across the diagonal axis of the plate to realize a loop current and excites the square-extensional mode^19^. Different from previous work, the device reported herein uses four separate excitation current tracks, which allows more flexible manipulation of four excitation currents in direction and frequency so that two vibration modes can be effectively excited for measurement of all 3-axis magnetic fields.

### Three-axis piezoelectric LFM

**Fig. 2:**
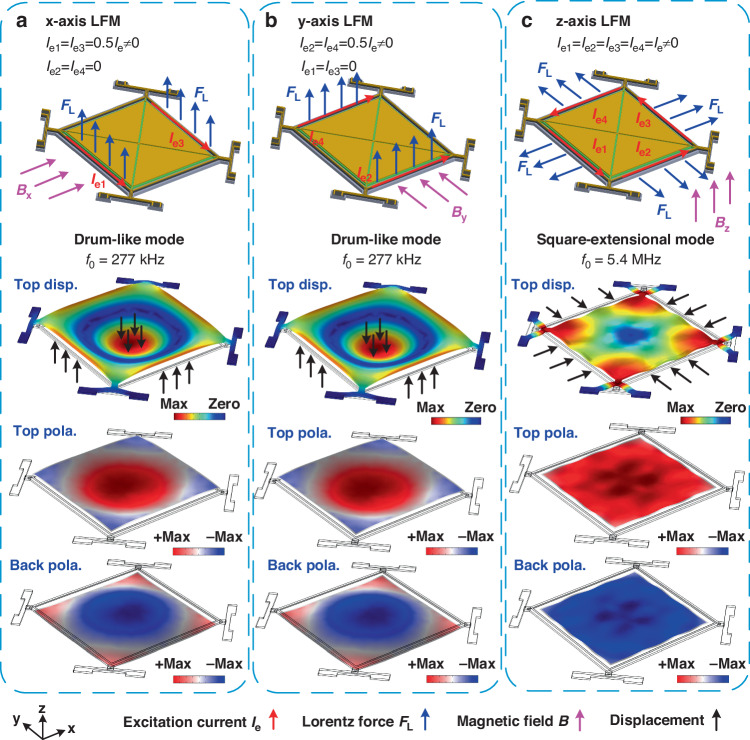
Three working statuses when the device is used as an x-, y- or z-axis LFM. In each status, the external magnetic field (*B*), applied alternating excitation current (*I*_*e*_) and generated Lorentz force (*F*_*L*_) are labeled with the displacement profile and polarization distribution of the target vibration modes obtained via finite element simulation: (**a**) x-axis LFM utilizes the drum-like mode excited by out-of-plane *F*_*L*_ resulting from two parallel current paths (*I*_*e1*_ and *I*_*e3*_); (**b**) y-axis LFM utilizes the drum-like mode excited by out-of-plane *F*_*L*_ resulting from two parallel current paths (*I*_*e2*_ and *I*_*e4*_); and (**c**) z-axis LFM utilizes the square-extensional mode excited by in-plane *F*_*L*_ resulting from one loop current path (*I*_*e1*_ = *I*_*e2*_ = *I*_*e3*_ = *I*_*e4*_)

Figure 2a–c illustrate the three working statuses when the device is used for x-axis, y-axis and z-axis LFMs, respectively. For x-axis LFM, as shown in Fig. 2a, the target drum-like mode is excited by the alternating Lorentz force (*F*_*L*_) resulting from the cross product of applied excitation currents (*I*_*e1*_ and *I*_*e3*_) and x-axis magnetic flux (*B*_*x*_), which can be described by the following expression:

1 $${F}_{L}={L}_{t}{I}_{e}\times {B}_{x}$$

where *L*_*t*_ denotes the length of the excitation current track. The generated Lorentz force modulates the out-of-plane vibration amplitude of the drum-like mode and yields an output current (*I*_*out*_) due to the direct piezoelectric effect. Thus, the magnitude of *B*_*x*_ can be deduced by measuring the output current^20^. Note that *I*_*e1*_ and *I*_*e3*_ should be applied with identical frequency around the resonant frequency of the target drum-like mode (277 kHz) so that Q-fold enhancement in sensitivity can be realized against far-from-resonance operation.

By setting *I*_*e2*_ and *I*_*e4*_ to zero, the device cannot respond to a y-axis magnetic field due to the absence of an excitation current normal to *B*_*y*_. In addition, the presence of a z-axis magnetic field has a minimal contribution to this out-of-plane drum-like mode since the resultant Lorentz forces from *B*_*z*_ and *I*_*e1*_ (or *I*_*e3*_) are in the plane. By utilizing the same working principle, the proposed LFM can be adopted to measure the y-axis magnetic field by switching on *I*_*e2*_ and *I*_*e4*_ and switching off *I*_*e1*_ and *I*_*e3*_, as shown in Fig. 2b. For the measurement of the z-axis magnetic field, the square-extensional mode, as reported in previous work^19^, is used when all four excitation currents are injected in a loop, as shown in Fig. 2c. Due to the symmetry of the square plate and the target square extensional mode, we implement this current loop configuration by electrically connecting four current tracks in series and applying one excitation current (*I*_*e1*_ = *I*_*e2*_ = *I*_*e3*_ = *I*_*e4*_). The configurations of the excitation currents in Fig. 2a–c differ from each other in direction or frequency. This difference leads to good immunity to cross interference.

In the proposed single-structure 3-axis piezoelectric LFMs, the mechanical vibration mode, excitation current path and device structure are considered comprehensively to measure the magnetic fields in three axes. Given that the excitation current paths are limited to the plane of device fabrication (i.e., the x–y plane), the out-of-plane vibration mode must be used for measuring in-plane magnetic fields (i.e., the x- and y-axis magnetic fields). In contrast, the in-plane vibration mode should be used for measuring the z-axis magnetic field. This suggests that both out-of-plane and in-plane vibration modes in a single structure are needed. For these vibration modes, the excitation current paths are designed at the sides of the plate structure, while the central area is implemented for the output. In this configuration, the drum-like mode is chosen among various out-of-plane modes because it exhibits decent electromechanical coupling and can be effectively excited by alternating forces on the sides. The in-plane square-extensional mode is chosen for the same reason. To facilitate the design, a square shape is chosen for the plate structure for two reasons. First, the out-of-plane drum-like modes in a square plate for x- and y-axis measurements have the same resonance frequency due to the symmetry of the square shape. Switches can be used to reconfigure different pairs of current paths for either x- or y-axis measurements. This solution requires only one interface circuit for the x- and y-axis LFMs, reducing the design complexity of the interface circuit. Second, the in-plane square-extensional mode for the z-axis measurement can be most effectively excited in the square plate. The same level of excitation current can be used along four sides of the square plate to generate equivalent Lorentz forces that match the square-extensional mode. The displacement profile and polarization distribution of the two target vibration modes are shown in Fig. 2, where both vibration modes are effectively actuated by side forces and generate charges from the central area. Additionally, the drum-like mode has a more concentrated area for charge generation than the square-extensional mode. This suggests that the former should have inferior performance to the latter, especially considering its much lower resonant frequency due to its flexural vibration mode nature.

## Results and discussion

### Electrical characterization of the two target vibration modes

**Fig. 3:**
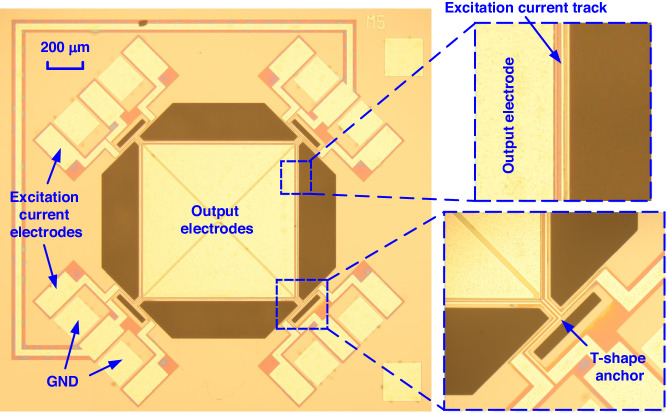
Optical micrograph of the fabricated LFM. The device comprises a square-plate AlN-on-Si resonator with a side length of 800 μm. The thicknesses of Al, AlN, SiO_2_, and Si layers are 1 μm, 500 nm, 200 nm and 10 μm, respectively. There are four triangular output electrodes on the plate with two electrodes connected diagonally on the chip. The square plate resonator is suspended by four T-shape anchors. There is an Al track running along each edge of the square plate, providing an electrical path for the excitation current. The excitation current is applied through two adjacent excitation current electrodes. There are three Al tracks on the T-shape anchor, providing two electrical paths for excitation currents and one for the output current

**Fig. 4:**
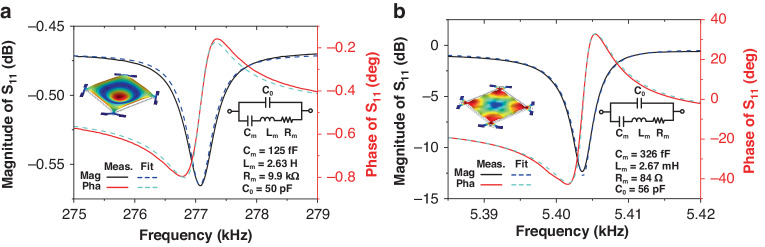
Measured magnitude and phase of S_11_ for the fabricated device. **a** Drum-like mode. **b** Ssquare-extensional mode. The extracted BVD model parameters of the two vibration modes are also provided

Figure 3 shows an optical micrograph of the LFM fabricated using a foundry AlN-on-Si process. A square-plate resonator with a side length of 800 μm is suspended by four T-shape anchors, which are clamped to the surrounding Si substrate. The suspended plate primarily comprises an Al layer for current tracks and electrodes, an AlN layer for piezoelectric transduction, a SiO_2_ layer for electrical isolation and a Si layer for acoustic cavity. The detailed physical dimensions of the device are listed in the supplementary file. To electrically characterize the two target vibration modes, we measure the reflection characteristics in terms of *S*_*11*_ at the output port of the fabricated device. Figure 4a, b show the measured *S*_*11*_ for the out-of-plane drum-like mode and in-plane square extensional mode, respectively. The drum-like mode exhibits a resonant frequency of 277 kHz, while the square extensional mode exhibits a resonant frequency of 5.44 MHz, which agrees with the simulated results shown in Fig. 2. Based on the measured *S*_*11*_, we extract the Butterworth van Dike (BVD) model parameters for both vibration modes, which are shown in Fig. 4.

### Electrical characterization for piezoelectric LFM

**Fig. 5:**
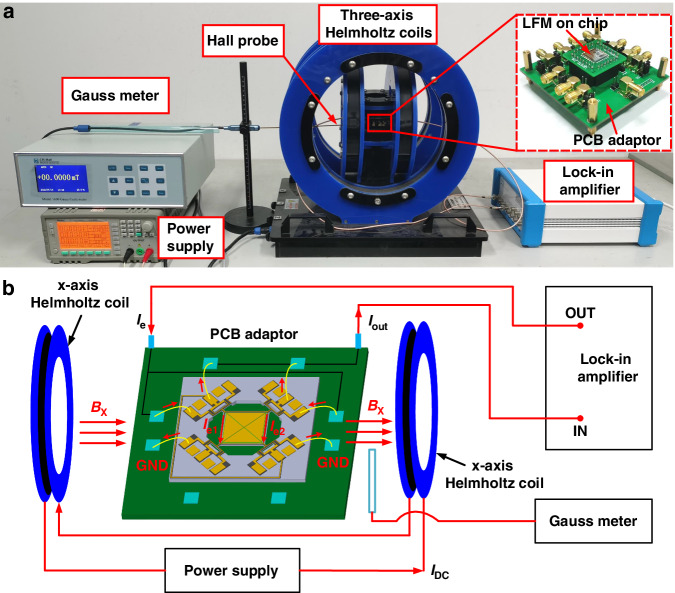
Electrical characterization assembly for the fabricated piezoelectric LFM. **a** Experimental setup. **b** Schematic for the assembly of the x-axis magnetic field measurement

**Fig. 6:**
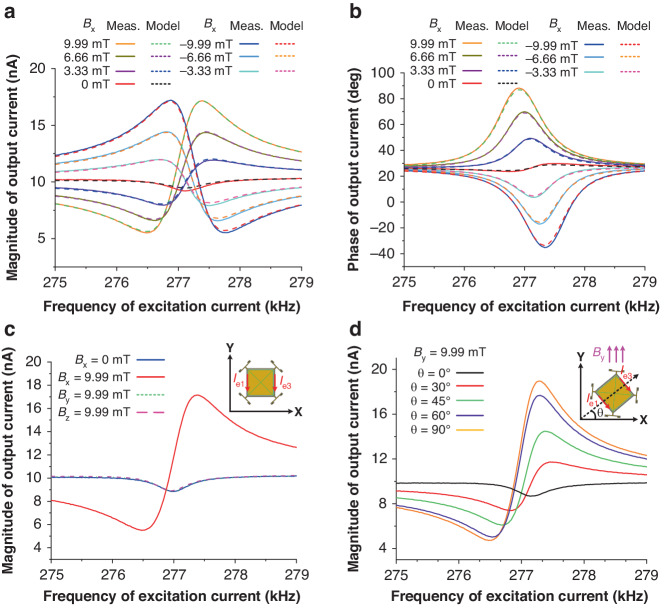
Measured output current (*I*_*out*_) for the x-axis LFM. **a**, **b** Measured magnitude and phase of the output currents when different levels of x-axis magnetic fields were applied. **c** Measured output currents when large y- and z-axis magnetic fields were applied to examine the cross-interferences immunity. **d** Measured output currents when the device was rotated under a fixed y-axis magnetic field of 9.99 mT. The excitation current (*I*_*e*_) was fixed at 662 μA during all these measurements

We used the experimental assembly shown in Fig. 5 to electrically characterize the proposed piezoelectric LFMs. Figure 6a, b show the measured magnitude and phase characteristics of the output currents (solid lines) when different levels of x-axis magnetic flux are applied (*B*_*x*_ = −9.99 mT ~ 9.99 mT). Figure 6a shows that the magnitude of the resonant signal increases as the absolute value of *B*_*x*_ increases. This result is expected since the Lorentz force scales with *B*_*x*_ when the excitation current is kept constant, as specified in Eq. (1). Additionally, an antiresonance is always present close to the resonance. This suggests that parasitic capacitive feedthrough coupling occurs from the input excitation current tracks to the output electrodes, which raises the baseline of the output current and introduces antiresonance. Figure 6b shows that the phase of the output current relative to the input excitation current changes gradually with increasing *B*_*x*_. The phase shift is smaller than 180°, which results from the presence of parasitic capacitive feedthrough. Interestingly, there is a small resonance peak in Fig. 6a when no magnetic field is applied (*B*_*x*_ = 0 mT). Since no magnetic field is applied, this resonance peak is believed not to result from the Lorentz force. Notably, this phenomenon was consistently observed in previously reported single-axis AlN-on-Si LFMs^7,19^, where the resonance peak at zero magnetic field was referred to as the offset. We believe that this offset is caused by the capacitive feedthrough, which provides a path for the input energy to leak from the input excitation current tracks to the output electrodes. Since this mechanism of energy leakage does not rely on the presence of an external magnetic field, the device is still driven into resonance by the leaked energy even when no magnetic field is applied. In the presence of an external magnetic field, the effect of Lorentz force starts to kick in and dominates the effect of the capacitive feedthrough in determining the resonance characteristics, as shown in Fig. 6a, b. To verify the abovementioned hypothesis, a novel equivalent circuit model considering the combined effect of the Lorentz force and capacitive feedthrough is proposed in the following, which yields reasonably good agreement with the measured results at different levels of external magnetic fields.

To examine the cross-interference immunity of the proposed LFM, we electrically characterize the device when a large magnetic field is applied to the nontarget axes. For the x-axis LFM, we apply large magnetic fields along the y- and z-axes (*B*_*y*_ = 9.99 mT or *B*_*z*_ = 9.99 mT). The measured output currents are plotted in Fig. 6c, where the measured results are shown to be almost the same as those for *B*_*x*_ = 0 mT. This finding suggests that the cross-interferences due to the y- and z-axis magnetic fields are much smaller than the offset. The measured results for small magnetic fields are provided in the supplementary file. The above measured results show that the proposed device, when operated as an x-axis LFM, exhibits good immunity to cross interferences from magnetic fields in the other two orthogonal axes. The same measurements are performed when the device is operated as y- or z-axis LFM, where good cross-interference immunity can be observed. Notably, the measured results shown in Fig. 6a–c are obtained when the device is effectively aligned to the target x-axis with the help of the alignment marker on the mounting platform. The impact from the y-axis magnetic field can be easily observed when the device rotates around the z-axis and deviates from the target x-axis, as shown in Fig. 6d.

### Equivalent circuit model

**Fig. 7:**
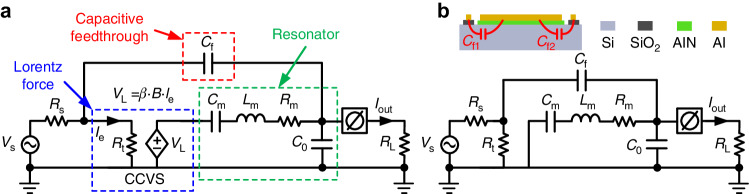
Equivalent circuit model for the proposed AlN-on-Si LFMs. **a** Circuit model. **b** Equivalent circuit for the case when a zero magnetic field is applied (*B* = 0 mT). In this case, the device is still driven into resonance by the leaked energy from the capacitive feedthrough path (represented by *C*_*f*_), which causes unwanted offset at the output

Although the measured magnitude of the output current changes with the applied magnetic field, as shown in Fig. 6a, the dependence of the output current resulting purely from the Lorentz force on the magnetic field cannot be directly observed due to interference from the effect of the capacitive feedthrough, which raises the baseline of the output current and introduces an unwanted offset. To effectively analyze the output of the device in response to different levels of magnetic field and gain understanding of the observed offset, we propose an equivalent circuit model for the fabricated piezoelectric LFM, as shown in Fig. 7a, which can capture both effects from the Lorentz force and capacitive feedthrough. In this model, *V*_*s*_ and *R*_*s*_ represent the voltage source and internal resistance of the lock-in amplifier, respectively. *R*_*t*_ denotes the total resistance of the excitation current tracks. A current-controlled voltage source (CCVS) is used to yield a voltage (*V*_*L*_) that is proportional to the Lorentz force. *β* is the ratio of *V*_*L*_ to the product of magnetic flux (*B*) and excitation current (*I*_*e*_). The mechanical resonance is described by the RLC series circuit (*C*_*m*_, *L*_*m*_, *R*_*m*_). *C*_*0*_ represents the static capacitance of the resonator. *C*_*f*_ denotes the capacitive feedthrough from the input excitation current tracks to the output electrodes. The phase shifter at the output port is used to account for the possible phase shifts introduced by the characterization assembly. The methods for obtaining these parameters are detailed in the supplementary materials. All the obtained parameters for the x-axis LFM are listed in Table 1. The extracted value of *R*_*m*_ (12.2 kΩ) is determined to be larger than the S_11_ measurement (9.9 kΩ), possibly because a new damping source is introduced by the second characterization setup, the effect of which can be lumped into *R*_*m*_. Figure 7b shows the equivalent circuit for the device at zero magnetic field (*B* = 0 mT), where the CCVS is electrically shorted at its output due to zero B. In such case, an electrical resonance can still be detected at the output because the device is driven by the leaked energy from the capacitive feedthrough path.

**Table 1:** Parameters for the equivalent circuit model of the proposed x-axis LFM

Parameters	Value	Parameters	Value
*V*_*s*_	100 mV	*C*_*f*_	1.5 pF
*R*_*s*_	145 Ω	*R*_*m*_	12.2 kΩ
*R*_*L*_	1 kΩ	*C*_*m*_	125 fF
*R*_*t*_	6 Ω	*L*_*m*_	2.63 H
*C*_*0*_	50 pF	*β*	−23 Ω/T

Fig. 6a, b plot the model fitting results (dashed lines) for the output currents at different levels of *B*_*x*_. Good agreement can be observed for the magnitude and phase characteristics of the output currents at different levels of *B*_*x*_, proving that the proposed circuit model can capture the electrical behavior of the device. However, a small discrepancy remains between the measured and model results for the case when *B*_*x*_ = 0 mT, as shown in Fig. 6a. This finding indicates that there may be additional offset effects that cannot be accounted for by the proposed model. Reportedly, thermal actuation due to the Joule heating effect by excitation current may be present in these devices^7^.

**Fig. 8:**
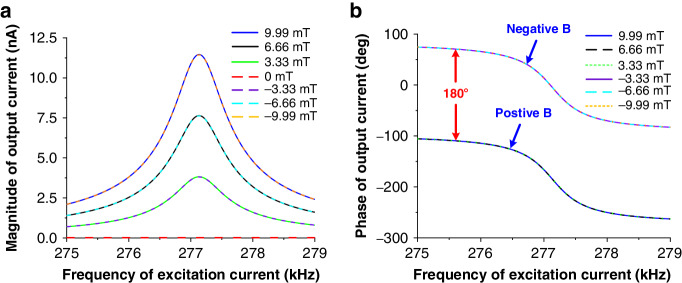
Output current (*I*_*out*_) from the pure effect of the Lorentz force using the proposed equivalent circuit model where *C*_*f*_ is removed. The excitation current (*I*_*e*_) is fixed at 662 μA during the measurement: **a** magnitude and **b** phase

**Fig. 9:**
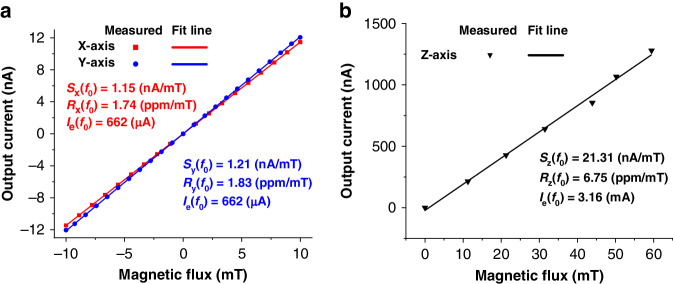
Obtained peak magnitude of the output current from the pure effect of the Lorentz force as a function of the external magnetic field. **a** Results for the x- and y-axis LFMs. **b** Results for the z-axis LFM. The calculated magnetic field sensitivities (*S*) and responsivities (*R*) along the three axes are denoted for comparison

By removing the capacitive feedthrough path (i.e., *C*_*f*_) in the proposed model, the effect of the Lorentz force can be readily extracted. Figure 8 shows the magnitude and phase of the output currents after removing the effect of the capacitive feedthrough. Figure 8a shows that the antiresonance effect disappears and that the magnitude of resonance in the output current increases with increasing *B*_*x*_. As expected, the magnitude of the output current remains unchanged when flipping the polarity of *B*_*x*_, but the phase of the output current shifts by 180°, as shown in Fig. 8b. This characteristic is important for identifying the direction of an external magnetic field. The same measurements are performed to measure the y-axis magnetic field, where *I*_*e2*_ and *I*_*e4*_ are used as the excitation currents instead of *I*_*e1*_ and *I*_*e3*_. The obtained results are similar to those shown in Figs. 6 and 8. This result is expected since the device appears identical along the x- and y-axes. The maximum magnetic field sensitivities *S*(*f*_*0*_) in both the x- and y-axes are obtained by finding the slopes of the best fit lines for the measured output currents at their resonances, as shown in Fig. 9a, where similar sensitivities are obtained for both axes. Due to the frequency limit of the lock-in amplifier, we use a vector network analyzer (N9913a) to perform z-axis magnetic field measurements with a characterization setup similar to that used in previous work^19^, and the obtained results are shown in Fig. 9b. Figure 9a, b show that the output currents of all three LFMs scale linearly with magnetic field. Given that the Lorentz force scales with the excitation current, as given in Eq. (1), the maximum responsivities *R*(*f*_*0*_) in the three axes are computed by dividing the sensitivities over the corresponding excitation currents for fair comparison. The obtained magnetic responsivities are 1.74 ppm/mT, 1.83 ppm/mT and 6.75 ppm/mT for the x-, y- and z-axes, respectively.

Table 2 shows a comparison of the performance of the proposed device against that of previously reported MEMS LFMs using AM modulation, where the responsivities of the proposed device are comparable to those of their capacitive counterparts without the constraint of vacuum operation. Notably, piezoelectric and capacitive LFMs exhibit comparable responsivities, although capacitive LFMs usually exhibit large Qs in vacuum. This occurs because piezoelectric LFMs have much larger electromechanical coupling to compensate for their lower Qs. A comparison of the different piezoelectric LFMs in Table 2 shows that the proposed z-axis piezoelectric LFM exhibits a similar responsivity to that of previous work. However, the responsivities of the proposed x- and y-axis LFMs are lower than those of previously reported single-axis piezoelectric LFMs^20^. This phenomenon arises because previous work uses corner-flapping mode in a square plate with straight beams as supporting anchors at the midpoints of the two sides, allowing effective actuation of the corner-flapping mode and yielding a large responsivity. However, such clamping boundary conditions cause large anchor losses for the square-extensional mode used herein for z-axis detection and great complexity in routing current paths for x- and y-axis detection. Thus, we conceive the drum-like mode with T-shape anchors at four corners, which benefits the actuation of the square-extensional mode and allows flexible reconfiguration for different current paths.

**Table 2:** Comparison of the performance characteristics of different MEMS LFMs using AM modulation

MEMS LFMs	Transduction Type	Detection Axis	Resonant Frequency	Responsivity (ppm/mT)	Operating Condition	Q Factor	Electromechanical Coupling (fC/nm)
Double-ended tuning fork^22^	Capacitive	z	49.3 kHz	0.22	Vacuum	100,000	0.05
Comb structure^12^	Capacitive	x or y	40.5 kHz	0.05	Vacuum	110	0.34
		z	107.4 kHz	0.13		310	0.62
Double-ended tuning fork^23^	Capacitive	x or y	20.55 kHz	2.45	Vacuum	1400	0.08
		z	46.96 kHz	1.41		10,000	0.22
Rectangular plate^24^	Piezoelectric	z	18.0 MHz	4.84	Air	1500	1417
Square plate^20^	Piezoelectric	x or y	160 kHz	12.02	Air	508	39.51
Disk^7^	Piezoelectric	z	6.3 MHz	12.55	Air	1946	1547
Square plate^19^	Piezoelectric	z	5.28 MHz	6.95	Air	1056	2844
**Square plate (This work)**	**Piezoelectric**	**x and y**	**277.1** **kHz**	**1.74/1.83**	**Air**	**470**	**51.4**
		**z**	**5.4** **MHz**	**6.75**		**1070**	**3100**

Bold text and values are related to the devices proposed by this work

Notably, both *S*(*f*) and *R*(*f*) peak at resonant frequencies of the target vibration modes, as specified by the AM mode operation principle. In practice, the operation frequency of the device can deviate from the resonant frequency due to factors such as changes in the ambient temperature, thus requiring knowledge of the responsivity as a function of the operation frequency. By analyzing the proposed equivalent circuit model, the dependence of the responsivity on the operation frequency can be obtained by the following equation:

2 $$R(f)=\left|\frac{{I}_{out}(f)}{{I}_{e}(f)\cdot B}\right|\approx \left|\frac{\beta }{{Z}_{r}(f)+{R}_{L}}\right|$$

where *Z*_*r*_(*f*) denotes the impedance of the RLC series circuit (*C*_*m*_, *L*_*m*_, *R*_*m*_). The approximation in Eq. (2) is made by considering that the impedances of *C*_*0*_ and *C*_*f*_ are usually much larger than that of *R*_*L*_. By using Eq. (2), the responsivity of the LFM can be quickly calculated when the motional parameters and load impedance are given.

### Excitation current and stability analysis

**Fig. 10:**
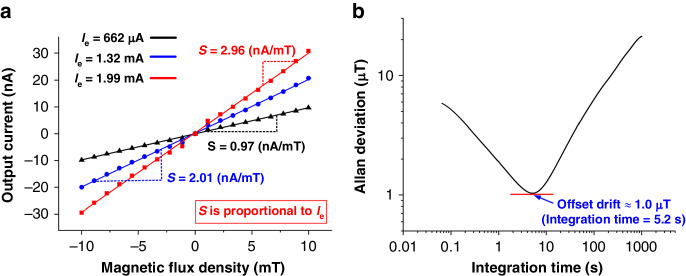
Output current and stability analysis for the x-axis LFM. **a** Measured output current (*I*_*out*_) of the x-axis LFM when different levels of excitation current (*I*_*e*_) are applied. **b** Measured Allan deviation to show the stability of the device

The operation of the proposed piezoelectric LFM hinges on the input AC excitation current. Figure 10a shows the measured output current of the x-axis LFM when different levels of excitation current are applied. The sensitivity of each device is scaled with the applied current level, as expected. Specifically, the sensitivity of the device can be enhanced by increasing the excitation current. However, a large excitation current results in additional power consumption and heat dissipation problems. Notably, the proposed LFMs have low input resistance values. For the proposed x- and y-axis LFMs, the input total resistance is 6 Ω due to the parallel connection of two 12 Ω Al tracks. For the proposed z-axis LFM, the input total resistance is 48 Ω due to the series connection of four 12 Ω Al tracks. Considering the current level for driving, the power consumption from the LFM alone should be considerably lower than that from the interface circuits.

To determine the stability, we measure the Allan deviation for the output current of the x-axis LFM with the excitation current maintained at 662 μA for 12 hours. Figure 10b shows the measured Allan deviation with a minimum instability of 1.0 μT under an integration time of 5.2 s. This stability performance is close to that of a previously reported piezoelectric LFM (0.7 μT with an integration time of 180 s), which operates in corner-flapping vibration mode with a resonant frequency at 160 kHz^20^. We measure the noise spectrum at the output port of the x-axis LFM using a lock-in amplifier, which is 1 pA/√Hz. This result translates to a noise density of 587 nT/√Hz with reference to an excitation current of 1 mA. Given that the calculated noise density for the x-axis LFM is only 306 nT/√Hz, the noise level of the x-axis LFM is dominated by the electronic noise of the lock-in amplifier. The calculation of noise density is detailed in the supplementary information.

### Current issues and potential solutions

The observed offset for the piezoelectric LMF upon application of a zero magnetic field is one issue that needs to be solved for these devices. Although we proposed an equivalent circuit model that can be used to remove the effect of this offset by postprocessing in this study, an on-chip real-time elimination or suppression method is still needed. Since the offset results from the capacitive feedthrough from input excitation current tracks to output electrodes, introducing a ground plane between the current tracks and output electrodes can help, but it was shown to have limited effect^7^. Another route to address this issue may be using a dummy twin structure that can be co-fabricated with the LMF and work as a compensator to mitigate the parasitic capacitive feedthrough^21^. The cost of this method is a doubled footprint for the whole device.

In this work, the proposed device is implemented in an open-loop scheme using a lock-in amplifier at room temperature. In future, it is expected to implement such piezoelectric LMFs using a closed-loop scheme, which is attractive for practical applications. One advantage of using a closed-loop scheme is to improve the temperature stability over a large temperature range. The advantage of LMFs operating in AM mode lies in the large sensitivity offered by the Q-amplification at the resonant frequency of the device. However, the resonant frequency changes with temperature, which makes it difficult to maintain the device at the target resonant frequency for the highest possible sensitivity. To address this issue, a closed-loop oscillator with an output frequency tracking the resonant frequency is desired. Notably, implementing a closed-loop scheme creates a new challenge in designing a highly stable oscillator that embeds piezoelectric LFM and low-noise interface circuits.

## Conclusions

In conclusion, we have demonstrated a single-structure 3-axis MEMS LFM using a square-plate AlN-on-Si resonator. The measurement of 3-axis magnetic fields in a single device is realized by manipulating the direction and frequency of the applied excitation currents toward two vibration modes. The obtained maximum magnetic responsivities are 1.74 ppm/mT, 1.83 ppm/mT and 6.75 ppm/mT in the three axes, which are comparable to those of state-of-the-art MEMS LFMs. This work reports an equivalent circuit model for analyzing the measured results and separating the two effects from the Lorentz force and capacitive feedthrough. The proposed equivalent circuit model is considered generally applicable to all LFMs based on AlN-on-Si resonators, which are often plagued by an inherent capacitive feedthrough.

## Materials and methods

### Fabrication process

The device was fabricated using a foundry AlN-on-Si process. The process began with a silicon-on-insulator (SOI) wafer with a 10 μm-thick silicon device layer. First, the wafer was annealed at high temperatures to drive the phosphorous dopant into the surface of the top silicon layer. A 200 nm-thick thermal oxide was grown and patterned for electrical isolation. Then, a 0.5 μm-thick AlN layer was deposited by reactive sputtering and patterned by wet etching. This process was followed by depositing 1 μm-thick Al, which was later patterned for current tracks and electrodes. The silicon device layer was etched via deep reactive ion etching (DRIE) to define the lateral dimensions of the resonator. Finally, the handling layer of the SOI wafer was etched through from the back side by DRIE, followed by wet etching of the buried oxide to release the resonator.

### Measurement setup

For electrical characterization of the two target vibration modes, we measured the magnitude and phase of the reflection coefficient *S*_*11*_ at the output port of the fabricated device using a vector network analyzer (Keysight N9914A). To electrically characterize the piezoelectric LFM, the fabricated die was mounted on a custom-designed printed circuit board (PCB) adaptor, where electrical connections to on-chip electrodes were made via wire bonds. Helmholtz coils were used to generate the magnetic field, the magnitude and direction of which were controlled by a DC power supply. The generated magnetic fields were recorded by a high-precision Gauss meter with a measurement range of ±100 mT and a resolution of 0.1 μT. For the measurement of the x- and y-axis magnetic fields, we placed the PCB adaptor in the center platform of the Helmholtz coils with the assistance of an alignment marker to ensure that the device was placed properly in the area with a uniform magnetic field. The frequency responses in the amplitude and phase of the device were measured using a digital lock-in amplifier (Zurich Instruments MFLI 500 kHz). The digital lock-in amplifier provided the input AC excitation currents to drive the device into operation, and it detected the output current. For the measurement of the z-axis magnetic field, we used a vector network analyzer (Keysight N9914A) to measure the transmission characteristics of the device in terms of the magnitude and phase of S_21_. All the measurements were performed at ambient pressure at room temperature.

### Supplementary information

supplementary_materials_clean
