# Supplementary material for: Single-structure 3-axis Lorentz force magnetometer based on an AlN-on-Si MEMS resonator
Source: Microsyst Nanoeng. 2024 May 9;10:58. doi: 10.1038/s41378-024-00696-3 (PMC11079019; doi:10.1038/s41378-024-00696-3)
Supplement: Supplementary file 1 — supplementary_materials_clean [file 41378_2024_696_MOESM1_ESM.pdf]

Supplementary material

1. Physical dimensions of proposed piezoelectric LFM

The physical dimensions of the proposed piezoelectric LMF are listed in Table S1:

Table S1 Physical dimensions of proposed piezoelectric LFM

Parameter	Material	Value (μm)
Side length of square plate	Si	800
Thickness of square plate		10
Length of excitation current track	Al	760
Width of excitation current track		4
Thickness of excitation current track		1
Thickness of isolation layer	SiO_2	0.2
Thickness of piezoelectric film layer	AlN	0.5

2. Procedure in obtaining parameters in Table 1

The parameters listed in Table 1 of manuscript were obtained using the methods detailed as follows:

(1) V_s and R_L were specified by the digital lock-in amplifier; R_t of 6Ω was measured across the excitation current tracks of the device by using a multi-meter; R_s was extracted using following procedure: (1) Set output voltage of lock-in amplifier to be 100mV; (2) Measure the output current when load resistor is 6Ω. The obtained measured current is 662μA. (3) Rs=100mV/662μA-6Ω= 145Ω.

(2) The motional parameters of the MEMS resonator (C_m, L_m, R_m) and its static capacitance (C_0) were extracted from the frequency response when by driving the resonator with piezoelectric actuation and detection using a vector network analyzer (N9913a).

(3) By fitting the proposed equivalent circuit model to the measured output current at no magnetic field, one can extract out the value of C_f, which represents the capacitive feedthrough. Also, the phase shift of the characterization setup can be account for by tuning the phase shifter.

(4) By fitting the proposed equivalent circuit model to the measured output current at non-zero magnetic fields, one can extract out the value of β.

3. Measured results for small levels of magnetic fields

Figure S1 shows the measured results for small levels of magnetic fields, where it can be seen that the curves in both magnitude and phase of output current change gradually with applied magnetic fields from 0 mT to 1 mT. It can be seen that applying small magnetic field of 0.1 mT in x axis induces notable change in output current compared to the case of no magnetic field applied, but we cannot distinguish the change in output current when applying large magnetic field of about 10 mT in y- and z-axis.

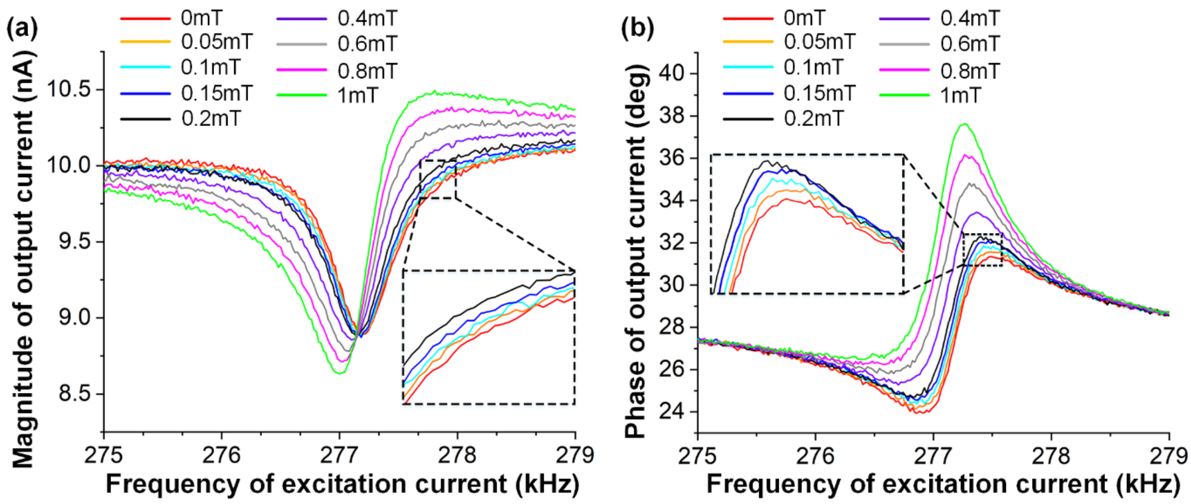

Fig. S1 Measured magnitude (a) and phase (b) of output current when small levels of x-axis magnetic fields were applied to x-axis LFM. The magnitude of excitation current (I_e) was fixed at 662 µA during the measurement.

4. Equivalent circuit model parameters for the z-axis LFM

The obtained of parameters for the equivalent circuit model of proposed z-axis LFM are listed in Table S2:

Table S2 Equivalent circuit model parameters for the z-axis LFM.

Parameters	Value	Parameters	Value
V_s	310 mV	C_f	1.5 pF
R_s	50 Ω	R_m	84 Ω
R_L	50 Ω	C_m	326 fF
R_t	48 Ω	L_m	2.67 mH
C_0	56 pF	β	-0.4 Ω/T

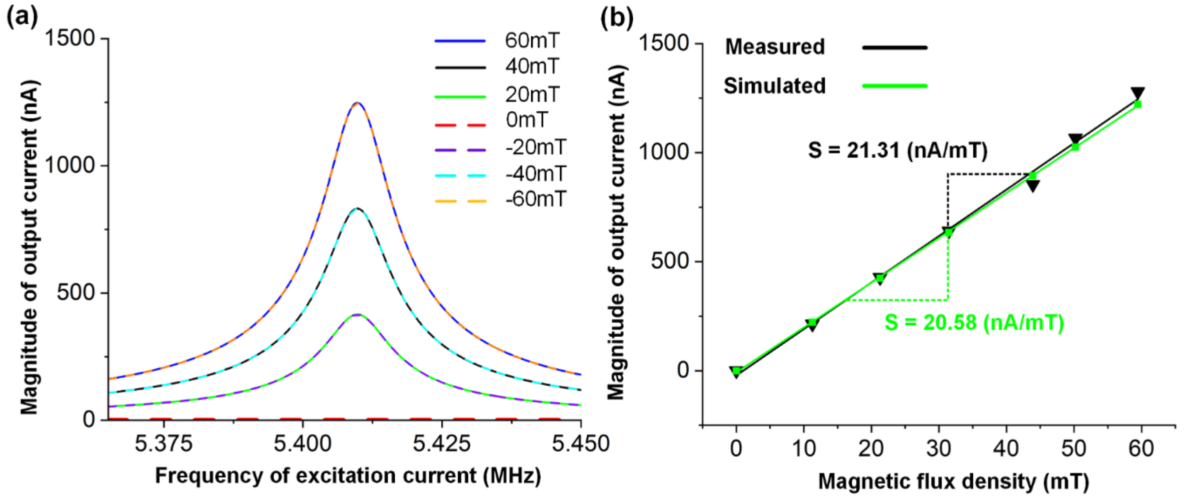

Fig. S2 Results for z-axis LFM. (a) Output current (I_{out}) from pure effect of Lorentz force for z-axis LFM using the proposed equivalent circuit model with parameters listed in Table S2; (b) Measured sensitivity for z-axis LFM compared to simulated results.

5. Detail noise analysis for the proposed piezoelectric LFM

We measured the noise density of output current of x-axis LFM, which was found to be 1 pA/√Hz. Dividing this noise density by the measured sensitivity (1.14 nA/mT) of x-axis LFM, we obtained a resolution of 877 nT/√Hz with reference to an excitation current of 662 μA. This translates to 587 nT/√Hz with reference to an excitation current of 1 mA. The equivalent thermomechanical noise limited B field resolution (B_n) of the square topology LFM within a 1 Hz bandwidth is calculated by the formula：

$$B_n = \frac{2}{L_t I_e}\sqrt{\frac{k k_B T}{(2\pi f_0)Q}}$$

where L_t denotes the length of excitation current track, I_e excitation current, k spring constant, k_B Boltzmann's constant, T absolute temperature, f_0 resonant frequency, Q quality factor. Therefore, we can obtain B_n for the x-axis LFM operating in drum-like mode, which is 306 nT/√Hz referenced to excitation current of 1 mA. This noise density is significantly smaller than the measured one (587 nT/√Hz), suggesting the noise level of x-axis LFM is dominated by the electronic noise of lock-in amplifier.
